# High serum levels of caspase-cleaved cytokeratin-18 are associated with malignant middle cerebral artery infarction patient mortality

**DOI:** 10.1186/s12883-018-1038-z

**Published:** 2018-03-24

**Authors:** Leonardo Lorente, María M. Martín, Antonia Pérez-Cejas, Luis Ramos, Mónica Argueso, Jordi Solé-Violán, Juan J. Cáceres, Alejandro Jiménez, Victor García-Marín

**Affiliations:** 1 Intensive Care Unit, Hospital Universitario de Canarias, Ofra s/n, La Laguna, -38320 Santa Cruz de Tenerife, Spain; 2 Intensive Care Unit, Hospital Universitario Nuestra Señora de Candelaria, Crta del Rosario s/n, -38010 Santa Cruz de Tenerife, Spain; 3 Laboratory Deparment, Hospital Universitario de Canarias, Ofra, s/n., La Laguna -, 38320 Tenerife, Spain; 4 Intensive Care Unit, Hospital General La Palma, Buenavista de Arriba s/n, -38713 Breña Alta, La Palma Spain; 5 Intensive Care Unit, Hospital Clínico Universitario de Valencia, Avda Blasco Ibáñez n°17-19, -46004 Valencia, Spain; 6 Intensive Care Unit, Hospital Universitario Dr. Negrín, CIBERES, Barranco de la Ballena s/n, -35010 Las Palmas de Gran Canaria, Spain; 7 Intensive Care Unit, Hospital Insular, Plaza Dr. Pasteur s/n, 35016 Las Palmas de Gran Canaria, Spain; 8 Research Unit, Hospital Universitario de Canarias, Ofra s/n. La Laguna, -38320 Santa Cruz de Tenerife, Spain; 9 Deparment of Neurosurgery, Hospital Universitario de Canarias, Ofra, s/n. La Laguna, 38320 Santa Cruz de Tenerife, Spain

**Keywords:** Caspase-cleaved cytokeratin-18, Cerebral infarction, Patients, Mortality

## Abstract

**Background:**

There have been found apoptotic changes in brain tissue samples from humans after cerebral ischemia. Caspase-cleaved cytokeratin (CCCK)-18 could appears in blood during apoptosis. High circulating levels of CCCK-18 have been associated with a poor prognosis in patients with cerebral process, such as traumatic brain injury and spontaneous cerebral hemorrhage. However, they have not been explored in patients with ischemic stroke. Thus, the aim of this study was to determine whether there is an association between serum CCCK-18 levels and mortality in patients with severe malignant middle cerebral artery infarction (MMCAI).

**Methods:**

This was an observational, prospective and multicentre study. We included patients with severe MMCAI. We considered MMCAI as severe when Glasgow Coma Scale (GCS) was lower than 9. We measured serum CCCK-18 levels at the diagnosis moment of the severe MMCAI.

**Results:**

We found that non-surviving severe MMCAI patients (*n* = 33) showed lower GCS and platelet count, and higher serum CCCK-18 levels than survivor ones (n = 33). We found an area under the curve (AUC) of serum CCCK-18 levels to predict 30-day mortality of 82% (95% CI = 71%–91%; *p* < 0.001). In the multiple logistic regression analysis was found that serum CCCK-18 levels were associated with 30-day mortality (OR = 1.023; 95% CI = 1.010–1.037; *p* = 0.001) after to control for platelet count and GCS.

**Conclusions:**

To our knowledge, this is the first series reporting data on serum CCCK-18 levels in ischemic stroke patients. The novel findings of our study were that non-surviving severe MMCAI patients had higher serum CCCK-18 levels than surviving patients, and that there is an association between high serum CCCK-18 levels and MMCAI patients mortality.

## Background

Ischemic stroke cause death, disability, and health resources consume [1]. In brain infarction appears cell death due to brain vasculature obstruction (which produces a restriction of oxygen and substrates for neurons) and due to apoptosis [2–7]. There have been found apoptotic changes in brain tissue samples from humans after cerebral ischemia [8–13].

Cytokeratins (CK) are proteins, until now named as CK-1 to CK-20, existing mainly in the intracytoplasmic cytoskeleton of epithelial tissue. During apoptosis CK-18 is cleaved at various sites by the action of caspases and appears caspase-cleaved cytokeratin (CCCK)-18, which could be released into the blood [14, 15].

Previously, there were found higher circulating CCCK-18 levels in patients with sepsis [16–20], liver diseases [21–25], and tumoral diseases [26, 27]. In addition, there was found an association between high circulating CCCK-18 levels and a poor prognosis of patients with different cerebral process, such as traumatic brain injury [28] and spontaneous cerebral hemorrhage [29, 30]. However, they have not been explored in patients with ischemic stroke. Thus, the aim of this study was to determine whether there is an association between serum CCCK-18 levels and mortality of patients with severe malignant middle cerebral artery infarction (MMCAI).

## Methods

### Design and subjects

This observational prospective multicentre study was carried with the written informed consent from patient legal guardians in 6 Intensive Care Units from Spain after the approval by the Institutional Review Board of all participanting hospitals: H. Insular from Las Palmas de Gran Canaria, H. General de La Palma from Breña Alta, H. Universitario de Canarias from La Laguna, Tenerife, H. Clínico Universitario de Valencia from Valencia, H. Universitario Dr. Negrín from Las Palmas de Gran Canaria, H.Universitario Nuestra Señora de Candelaria from Santa Cruz de Tenerife.

We included patients with severe malignant middle cerebral artery infarction (MMCAI). We estimated the severity of MMCAI according to Glasgow Coma Scale (GCS) [31], and we defined a MMCAI as severe when GCS ≤ 8. We excluded patients with age less than 18 years, pregnancy, inflammatory or malignant disease, intracerebral hemorrhage or subarachnoid hemorrhage.

Previously, we determined in some of those patients serum levels of biomarkers related with inflammation, coagulation and oxidation such as substance P [32], soluble CD154 [33] and malondialdehyde [34]. The aim of the current research was to determine serum levels of a biomarker related with apoptosis, such as CCCK-18, in 66 patients with severe MMCAI.

### Variables recorded

We recorded the following variables in each patient: age, sex, decompressive craniectomy, sodium, temperature, leukocytes, glycemia, pressure of arterial oxygen (PaO2), fraction inspired oxygen (FI0_2_), creatinine, bilirubin, hemoglobin, lactic acid, GCS, platelets, international normalized ratio (INR), fibrinogen, activated partial thromboplastin time (aPTT), Acute Physiology and Chronic Health Evaluation II (APACHE II) score [35]. The end-point study was 30-day mortality.

### Blood sample collection and serum CCCK-18 analysis

Serum blood samples were collected at the moment of the MMCAI diagnosis to measure serum CCCK-18 levels. All determinations were performed at the Laboratory Department of the Hospital Universitario de Canarias (La Laguna, Tenerife, Spain). We determine serum CCCK-18 levels by enzyme-linked immunosorbent assay (ELISA) using M30 Apoptosense® ELISA kit (PEVIVA AB, Bromma, Sweden). The intra-assay coefficient of variation (CV), inter-assay CV, and detection limit assay were < 10%, < 10% and 25 u/L respectively.

### Statistical methods

Continuous and categorical variables were reported as medians (and interquartile ranges) and frequencies (and percentages) respectively. Continuous and categorical variables were compared between groups using Wilcoxon-Mann-Whitney test and chi-square test respectively. We carried out a multiple logistic regression to anlyze the association between serum CCCK-18 levels and mortality at 30 days after to control for platelet count and GCS. We calculated Odds Ratio and its 95% confidence intervals (CI) to measure the clinical impact of predictor variables. We performed receiver operating characteristic (ROC) curve to determine the prediction capacity of serum CCCK-18 levels for mortalty at 30 days. We constructed 30-day mortality Kaplan-Meier curves of patiens with higher and lower serum CCCK-18 levels than 298 u/L. Youden J index was used for the selection of 298 u/L as the optimal prognostic cut-off value of serum CCCK-18 level. All *p-*values lower than 0.05 were considered statistically significant. We performed statistical analyses using SPSS 17.0 (SPSS Inc., Chicago, IL, USA), LogXact 4.1, (Cytel Co., Cambridge, MA), and NCSS 2000 (Kaysville, Utah).

## Results

A total of 33 of 66 patients (50.0%) with severe MMCAI died within 30-day diagnosis. We did not find statistically significant differences between non-surviving and surviving patients in age, sex, descompressive craniectomy, temperature, sodium, PaO2, PaO2/FI02 ratio, leukocytes, lactic acid, INR, hemoglobin, glycemia, fibrinogen, creatinine, bilirubin, aPTT, and APACHE-II score. Although we found that non-surviving MMCAI patients showed lower GCS and platelet count, and higher serum CCCK-18 levels than survivor ones (Table 1).

**Table 1:** Clinical and biochemical characteristics of MMCAI patients according to 30-day survival

	Survivors (*n* = 33)	Non-survivors (n = 33)	*P* value
Age (years) - median (p 25–75)	59 (47–68)	64 (54–70)	0.30
Gender female - n (%)	14 (42.4)	13 (39.4)	0.99
Arterial hypertension - n (%)	19 (57.6)	16 (48.5)	0.62
Diabetes mellitus - n (%)	4 (12.1)	9 (27.3)	0.22
Chronic renal failure - n (%)	2 (6.1)	2 (6.1)	0.99
COPD - n (%)	1 (3.0)	1 (3.0)	0.99
Heart failure - n (%)	1 (3.0)	1 (3.0)	0.99
Haemorrhagic transformation - n (%)	7 (21.2)	6 (18.2)	0.99
Decompressive craniectomy – n (%)	9 (27.3)	6 (18.2)	0.56
Temperature (°C) - median (p 25–75)	36.4 (35.8–37.0)	37.0 (36.0–37.4)	0.19
Sodium (mEq/L)- median (p 25–75)	139 (137–145)	140 (139–146)	0.41
Platelets - median×10^3^/mm^3^ (p 25–75)	214 (170–280)	170 (131–212)	0.008
PaO2 (mmHg) - median (p 25–75)	137 (104–207)	114 (86–153)	0.26
PaO2/FI0_2_ ratio - median (p 25–75)	300 (197–372)	248 (184–330)	0.22
Leukocytes-median×10^3^/mm^3^ (p 25–75)	12.5 (9.5–17.0)	13.9 (9.3–21.4)	0.43
Lactic acid (mmol/L)-median (p 25–75)	1.30 (0.90–1.70)	1.40 (1.00–2.10)	0.25
INR - median (p 25–75)	1.09 (1.01–1.20)	1.20 (1.05–1.31)	0.10
Hemoglobin (g/dL) - median (p 25–75)	12.2 (11.4–14.4)	13.7 (11.0–15.0)	0.78
Glycemia (g/dL) - median (p 25–75)	128 (100–170)	135 (105–160)	0.99
GCS score - median (p 25–75)	7 (6–8)	6 (3–7)	0.01
Fibrinogen (mg/dl) - median (p 25–75)	440 (335–494)	419 (311–631)	0.83
Creatinine (mg/dl) - median (p 25–75)	0.80 (0.60–1.15)	1.00 (0.76–1.28)	0.12
CCCK-18 (u/L) - median (p 25–75)	238 (160–290)	321 (279–351)	< 0.001
Bilirubin (mg/dl) - median (p 25–75)	0.70 (0.40–0.95)	0.70 (0.33–1.10)	0.86
aPTT (seconds) - median (p 25–75)	28 (26–30)	27 (26–32)	0.77
APACHE-II score - median (p 25–75)	20 (16–25)	22 (19–27)	0.09

*COPD*  Chronic Obstructive Pulmonary Disease, *P 25–75*  Percentile 25th–75th, *PaO*_2_  Pressure of arterial oxygen/fraction inspired oxygen, *FIO*_2_  Pressure of arterial oxygen/fraction inspired oxygen, *INR*  International normalized ratio, *GCS*  Glasgow Coma Scale *aPTT*  activated partial thromboplastin time, *APACHE II*  Acute Physiology and Chronic Health Evaluation, *CCCK* Caspase-cleaved cytokeratin

**Fig. 1:**
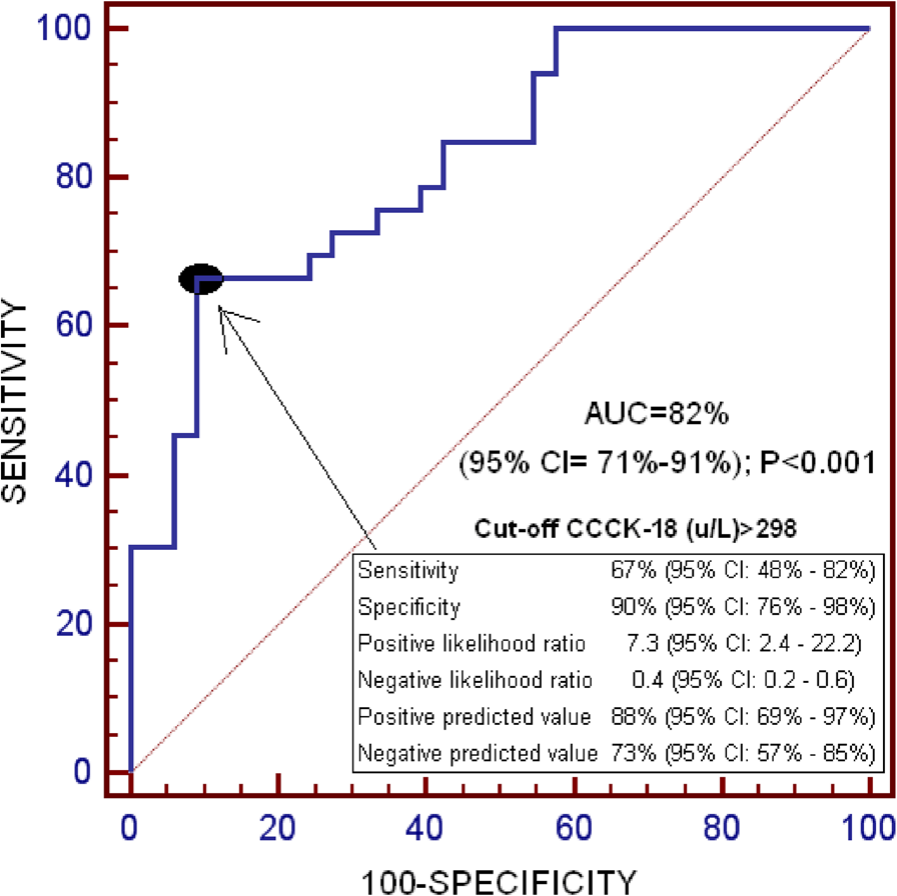
Receiver operation characteristic analysis using serum caspase-cleaved cytokeratin (CCCK)-18 levels as predictor of mortality at 30 days

**Fig. 2:**
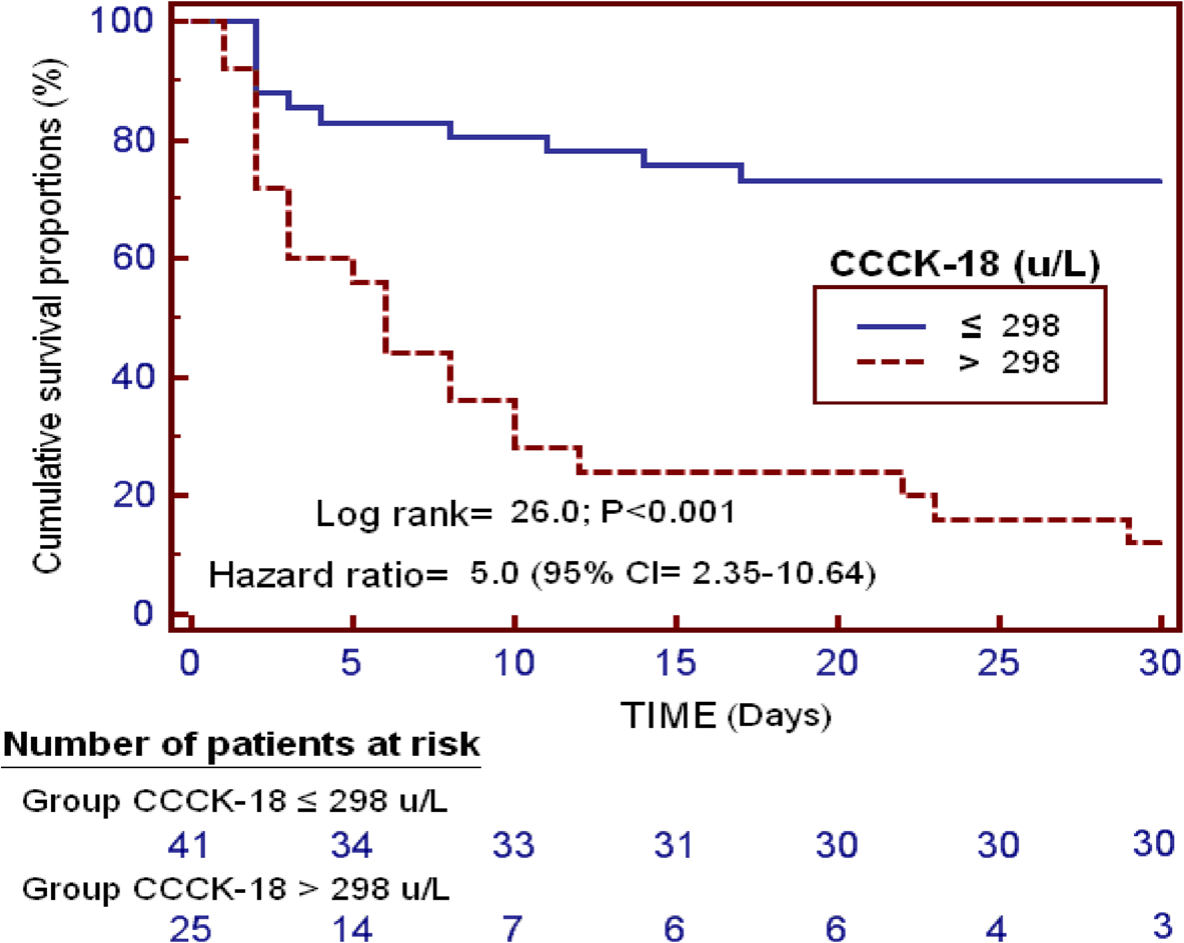
Survival curves at 30 days using serum levels of caspase-cleaved cytokeratin (CCCK)-18 higher or lower than 298 u/L

We found an area under the curve of serum CCCK-18 levels to predict mortality at 30 days of 82% (95% CI = 71%–91%; *p* < 0.001) (Fig. 1). In survival analysis was found that patients with serum CCCK-18 levels higher than 298 u/L showed a higher risk of mortality at 30 days (Hazard ratio = 5.0; 95% CI = 2.35–10.64; p < 0.001) than patients showed lower levels (Fig. 2).

In the multiple logistic regression was found that serum CCCK-18 levels were associated with mortality at 30 days (OR = 1.023; 95% CI = 1.010–1.037; *p* = 0.001) after to control for platelet count and GCS (Table 2).

**Table 2:** Multiple logistic regression analysis to predict 30-day mortality

Variable	Odds Ratio	95% Confidence Interval	*P*
Serum CCCK-18 levels (u/L)	1.023	1.010–1.037	0.001
Glasgow Coma Scale (points)	0.769	0.534–1.105	0.16
Platelet count (each 1000/mm^3^)	0.987	0.975–0.998	0.02

*CCCK*  Caspase-cleaved cytokeratin

## Discussion

To our knowledge, this is the first series reporting data on serum CCCK-18 levels in ischemic stroke patients. The novel findings of our study were that non-surviving severe MMCAI patients had higher serum CCCK-18 levels than surviving patients, and that there is an association between high serum CCCK-18 levels and MMCAI patients mortality.

Previously there has been found apoptotic changes in brain tissue samples from humans after cerebral ischemia [8–13]. However, the association between high serum CCCK-18 levels and MMCAI patients mortality found in our study is a novel finding. Those findings are in consonance with those of previous studies, due to that there is be found an association between high serum CCCK-18 levels and poor prognosis of patients with traumatic brain injury [28], acute spontaneous intracerebral haemorrhage [29] and aneurysmal subarachnoid hemorrhage [30].

The interpretation of all those findings is uncertain. Cytokeratin-18 exists mainly in the intracytoplasmic cytoskeleton of epithelial tissue and during apoptosis citokeratin-18 is cleaved by caspases and appears as CCCK-18 into the blood [14, 15]. Then the question about the origen of CCCK-18 in patients with traumatic brain injury [28], spontaneous cerebral hemorrhage [29, 30], and cerebral infarction (our current study) arise now. There is two posible splanations for that question. First, that there is cytokeratin-18 in brain; and this has been found in a study of patients with pituitary adenomas [36], and in a study of rats with glioma [37]. In the study by Luiciani et al. was found CCCK-18 in cell extracts of patients with pituitary adenomas, and the use of octreotide induced apoptosis in cells of growth hormone-secreting tumors assessed by the increased of CCCK-18 in cell extracts [36]. In the study by Adri et al was found CCCK-18 in cell extracts of glioma from rats, and the use of *Parmelia sulcata* Taylor (one of the most common lichens that lives mainly in the bark of the trees) induced apoptosis in cell tumors assessed by the increased of CCCK-18 in cell extracts [37]. Second, that MMCAI may cause a systemic inflammatory response syndrome (SIRS), and this could activate sistemic cellular apoptosis. In fact, there are studies reporting SIRS after cerebral infarction [38–40], and in SIRS appears different pro-inflammatory cytokines [41] that could activate apoptosis [2–7].

The administration of some antiapoptotic agents in ischemic cerebral animal models have reduced brain apoptosis degree and functional deficits [42–44].

Some limitations of our study should be recognized. First, data about the evolution of circulating CCCK-18 concentrations during the evolution of non-surviving and surviving patients were not reproted. Second, data about serum CCCK-18 levels in healthy controls were not reported; although, the objective of our study was to determine whether there is an association between serum CCCK-18 levels and mortality in MMCAI patients and was not to determine whether there is an increase of serum CCCK-18 levels in MMCAI patients. Third, we have not explored apoptosis in cerebral samples; although, the objective of our study was to determine whether apoptosis is associated with mortality of MMCAI patients using a technique easily reproducible by other researchers. Fourth, we have not data about how many patients were excluded from the study and the exclusion motivation.

## Conclusions

To our knowledge, this is the first series reporting data on serum CCCK-18 levels in ischemic stroke patients. The novel findings of our study were that non-surviving severe MMCAI patients had higher serum CCCK-18 levels than surviving patients, and that there is an association between high serum CCCK-18 levels and MMCAI patients mortality.

## Abbreviations

APACHE: Acute Physiology and Chronic Health Evaluation
CCCK: Caspase-cleaved cytokeratin
FIO_2_: Fraction inspired of oxygen
GCS: Glasgow Coma Scale
ICU: Intensive Care Unit
INR: International normalized ratio
ISS: Injury Severity Score
PaO_2_: Pressure of arterial oxygen
